# Multi-Shaped Ag Nanoparticles in the Plasmonic Layer of Dye-Sensitized Solar Cells for Increased Power Conversion Efficiency

**DOI:** 10.3390/nano7060136

**Published:** 2017-06-04

**Authors:** Da Hyun Song, Ho-Sub Kim, Jung Sang Suh, Bong-Hyun Jun, Won-Yeop Rho

**Affiliations:** 1 Department of Chemistry, Seoul National University, Seoul 151-747, Korea; songssi87@snu.ac.kr (D.H.S.); hosub@snu.ac.kr (H.-S.K.); jssuh@snu.ac.kr (J.S.S.); 2 Department of Bioscience and Biotechnology, Konkuk University, Seoul 143-701, Korea

**Keywords:** Ag nanoparticle, dye-sensitized solar cell, plasmonic layer

## Abstract

The use of dye-sensitized solar cells (DSSCs) is widespread owing to their high power conversion efficiency (PCE) and low cost of manufacturing. We prepared multi-shaped Ag nanoparticles (NPs) and introduced them into DSSCs to further enhance their PCE. The maximum absorption wavelength of the multi-shaped Ag NPs is 420 nm, including the shoulder with a full width at half maximum (FWHM) of 121 nm. This is a broad absorption wavelength compared to spherical Ag NPs, which have a maximum absorption wavelength of 400 nm without the shoulder of 61 nm FWHM. Therefore, when multi-shaped Ag NPs with a broader plasmon-enhanced absorption were coated on a mesoporous TiO_2_ layer on a layer-by-layer structure in DSSCs, the PCE increased from 8.44% to 10.22%, equivalent to an improvement of 21.09% compared to DSSCs without a plasmonic layer. To confirm the plasmon-enhanced effect on the composite film structure in DSSCs, the PCE of DSSCs based on the composite film structure with multi-shaped Ag NPs increased from 8.58% to 10.34%, equivalent to an improvement of 20.51% compared to DSSCs without a plasmonic layer. This concept can be applied to perovskite solar cells, hybrid solar cells, and other solar cells devices.

## 1. Introduction

In the near future, we predict that photovoltaic cells will be utilized in numerous fields, such as in mobile commerce or in the development of integrated photovoltaics (BIPVs) and vehicles. Moreover, photovoltaic cells are an essential component in Smart Grids utilized commonly these days. To realistically apply solar energy in Smart Grids, photovoltaic cells are required to have features including transparency, flexibility, light weight, low cost, and high power conversion efficiency (PCE). In terms of low cost and light weight, organic, inorganic, and hybrid materials have brighter prospects than semiconductors. Dye-sensitized solar cells (DSSCs) whose structure consists of a photoanode (organic dye and mesoporous TiO_2_ films on fluorine-doped tin oxide (FTO) glass), electrolyte (iodide/triiodide, I^−^/I_3_^−^), and a counter-electrode (platinum (Pt)-coated FTO glass) are used widely because of their strengths [1,2,3]. The DSSCs have several advantages, showing higher performance with relatively lower cost, lower handling expenses, lower strength of optical and incidence angles, higher mechanical durability, lighter weight, and more aesthetically pleasing and transparent design. Many approaches, including improvement of light-harvesting and carrier collection, are available to further improve the PCE of the DSSCs [4,5,6,7,8]. However, such improvements might come at a cost, adversely affecting other properties, such as charge separation and, ultimately, a reduction in the PCE [9]. 

Plasmonic materials could assist in absorbing more light from subwavelength antennas or in generating plasmon polaritons from incident light by trapping light energy on metal nanoparticles (NPs) or scattering the light. For this reason, the PCE would be improved due to increases in the short-circuit current density (*J_sc_*) in solar cells. 

The plasmonic effect of metal (e.g., Ag and Au) NPs can improve light harvesting [10,11,12]. When plasmonic metal NPs are applied to the DSSCs, light-harvesting or carrier collection can be improved with a minimal negative impact on other material properties [13,14,15]. Since the PCE is highly influenced by the type of plasmonic materials and configuration of the DSSCs, tuning the plasmonic properties in DSSCs can enhance the PCE [10,11,12,13,14,15,16]. Recently, three different types of Ag NPs were mixed, covering the absorption wavelength of N719 dye. The mixture was used in high-efficiency DSSCs, and exhibited the highest PCE for plasmonic DSSCs [17]. Furthermore, an Au and Ag NP-based double-layered composite film structure—the bottom layer of which consisted of TiO_2_ and Ag and the top layer of TiO_2_ and Au—showed enhanced efficiency [18]. Unfortunately, it is difficult to prepare composite films—i.e., various shapes, sizes and types of metal NPs must be synthesized, which are later mixed with semimetals to maintain their paste state.

In this study, multi-shaped Ag NPs were prepared and applied to DSSCs to enhance their PCE. Prepared multi-shaped Ag NPs formed various shapes, including spherical, rod, and triangular structures, which exhibited broader absorption wavelengths than that of the spherical Ag NPs. Thus, absorption of the plasmonic layer based on multi-shaped Ag NPs combined with Au NPs covered the absorption range of N719 dye. To study the plasmon-enhanced effect of the multi-shaped Ag NPs, the multi-shaped Ag NPs were applied in a layer-by-layer structure and in a composite film structure on DSSCs.

## 2. Results and Discussion

**Figure 1:**
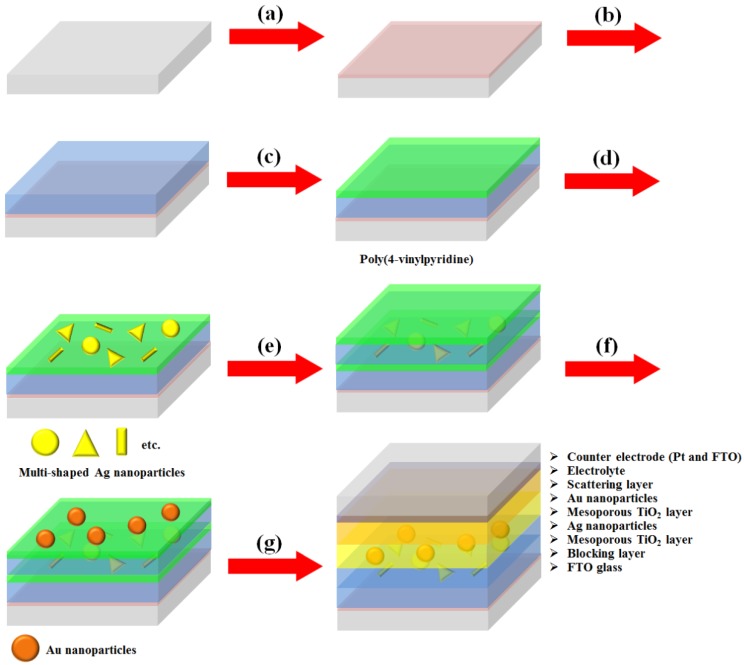
Fabrication process of DSSCs based on the layer-by-layer structure with multi-shaped Ag and Au nanoparticles (NPs): (**a**) coating of the TiO_2_ blocking layer; (**b**) coating of the mesoporous TiO_2_ layer on the TiO_2_ blocking layer; (**c**) coating of the poly(4-vinylpyridine) on the mesoporous TiO_2_ layer;(**d**) immobilization of the multi-shaped Ag NPs; (**e**) recoating of the mesoporous TiO_2_ layer and poly(4-vinylpyridine); (**f**) immobilization of the Au NPs; and (**g**) fabrication of the DSSC.

We synthesized multi-shaped Ag NPs with broad plasmon-enhanced absorption and applied them to DSSCs based on N719 dye. We fabricated the DSSCs based on a layer-by-layer structure with multi-shaped Ag and Au NPs by poly (4-vinylpyridine) (P4VP) (Figure 1) [19]. First, the TiO_2_ blocking layer was coated on the fluorine-doped tin oxide (FTO) glass (Figure 1a). The mesoporous TiO_2_ layer was coated using the doctor-blade technique, which became the first TiO_2_ layer (Figure 1b). The P4VP was coated on a mesoporous TiO_2_ layer (Figure 1c). Then, multi-shaped Ag NPs were immobilized on the first TiO_2_ layer coated with the P4VP, which became the first plasmonic layer (Figure 1d). The mesoporous TiO_2_ layer was also coated using the doctor-blade technique, which became the second TiO_2_ layer, and P4VP was recoated on the second TiO_2_ layer (Figure 1e). Subsequently, Au NPs were immobilized on the second TiO_2_ layer with the P4VP, which became the second plasmonic layer (Figure 1f). Finally, the scattering layer was coated using the doctor-blade technique, and the DSSCs were fabricated with an electrolyte and a counter-electrode after the removal of the P4VP by sintering (Figure 1g). To confirm that the layer-by-layer structure was maintained in DSSCs, the cross-section image of SEM was obtained from photoanode of DSSC. The thicknesses of the first TiO_2_ layer (Figure S1, bottom), second TiO_2_ layer (Figure S1, middle), and the scattering layer (Figure S1, top) were 5.44 μm, 4.83 μm, and 5.67 μm, respectively. The existence of Ag and Au NPs in the TiO_2_ film was confirmed by the EDX spectra, as shown in Figure S2. 

**Figure 2:**
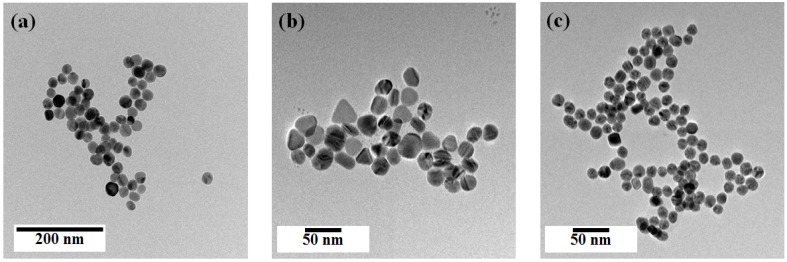
TEM images of (**a**) spherical Ag NPs, (**b**) multi-shaped Ag NPs, and (**c**) Au NPs.

Figure 2 shows transmittance electron microscope (TEM) images of the synthesized spherical Ag NPs, multi-shaped Ag NPs, and Au NPs. The spherical Ag NPs (average size: 29 ± 1.8 nm) were prepared following a well-known seed-growth method (Figure 2a). The multi-shaped Ag NPs were prepared following a simple one-step seed-mediated process that yielded spherical, rod, and triangle shapes (Figure 2b). The Au NPs (average size: 19 ± 1.5 nm) were prepared following the Turkevich method (Figure 2c). The size distribution histograms of Ag and Au NPs are as shown in Figure S3. 

**Figure 3:**
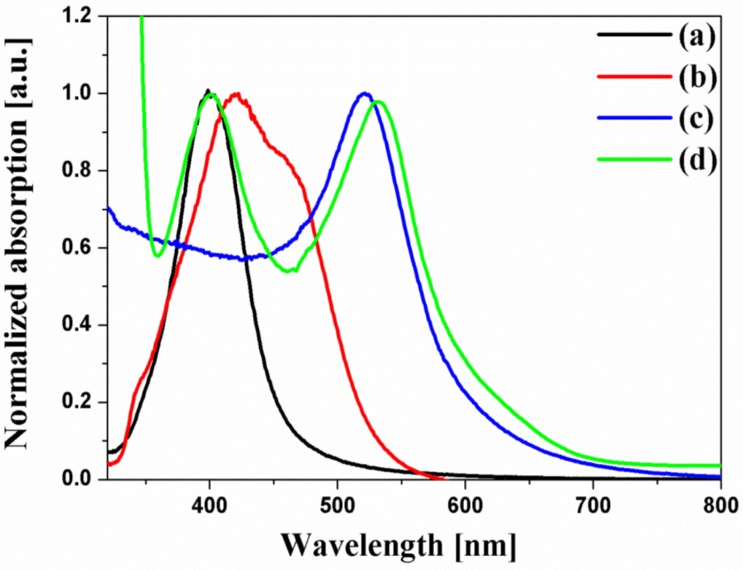
Normalized UV-VIS spectra of (**a**) spherical Ag NPs, (**b**) multi-shaped Ag NPs, (**c**) Au NPs, and (**d**) N719 dye.

Figure 3 shows the UV-VIS spectra of the spherical Ag NPs, multi-shaped Ag NPs, Au NPs, and N719 dye. N719 dye had two absorption bands which are an absorption band at higher energy (393 nm) with a 107 nm FWHM, λ_1_ and an absorption band at lower energy (533 nm) with a 108 nm FWHM, λ_2_. The UV-VIS spectra of the spherical Ag NPs exhibited a maximum absorption wavelength, λ_max_, of about 400 nm, which was matched by the λ_1_ of the N719 dye, and the FWHM of the spherical Ag NPs (61 nm) was narrower than that of the λ_1_ of the N719 dye. Meanwhile, the UV-VIS spectra of multi-shaped Ag NPs exhibited a λ_max_ of about 420 nm, which overlapped with the λ_1_ of the N719 dye, and the FWHM of multi-shaped Ag NPs (121 nm) was broader than the λ_1_ of the N719 dye. To study the plasmon-enhanced effect, the spherical Ag NPs (λ_max_ of about 400 nm) or multi-shaped Ag NPs (λ_max_ of about 420 nm) were introduced on the DSSCs as the first plasmonic layer overlapped with the λ_1_ of the N719 dye. Moreover, to improve the PCE of the DSSCs, Au NPs were introduced on the DSSCs as the second plasmonic layer. The size of the Au NPs was about 19 nm, and the λ_max_ was 520 nm, which overlapped with the λ_2_ of the N719 dye. Although 5 nm Au NPs exhibited better results in DSSCs [20,21], the λ_max_ of plasmonic NPs should also be considered. The λ_max_ of Au (19 nm) and Ag (29 nm) NPs are 524 nm and 400 nm, respectively, which show a quiet significant spectral overlap with two absorption bands of N719 dye (533 nm and 393 nm). In a layer-by-layer structure, the localized surface plasmon resonance (LSPR) field distributions of Au and Ag NPs under the excitation at their plasmon wavelengths were simulated by the finite difference time-domain (FDTD) method, as shown in Figure S4. The simulation showed the existence of the local field enhancement of Au and Ag NPs in the layer-by-layer structure.

**Figure 4:**
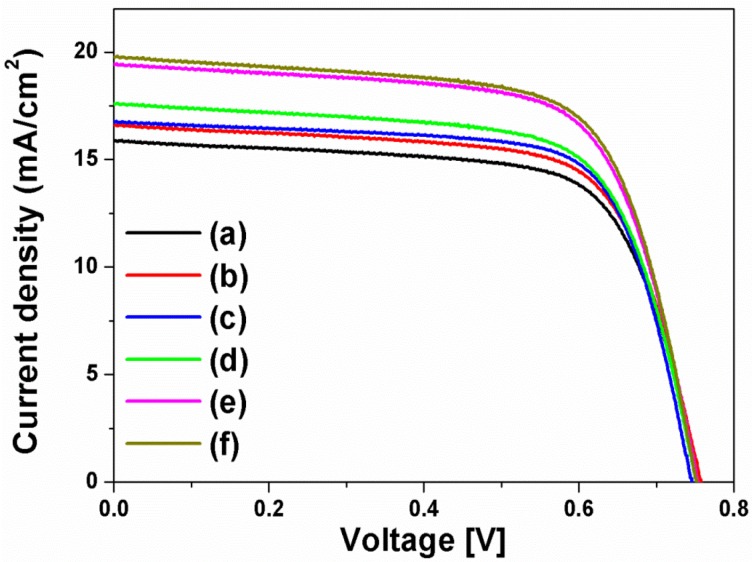
Current density-voltage curves of DSSCs based on a layer-by-layer structure: (**a**) without metal NPs, (**b**) with spherical Ag NPs, (**c**) with multi-shaped Ag NPs, (**d**) with Au NPs, (**e**) with spherical Ag and Au NPs, and (**f**) with multi-shaped Ag and Au NPs.

The current density-voltage (I-V) curves in Figure 4 were obtained from the DSSCs based on the layer-by-layer structure without or with spherical Ag NPs, multi-shaped Ag NPs, or Au NPs; the photovoltaic parameters are summarized in Table 1. To study the first plasmon-enhanced effect with spherical Ag NPs or multi-shaped Ag NPs, except the second plasmonic layer, three types of DSSCs based on the layer-by-layer structure were fabricated without metal NPs, with spherical Ag NPs, or with multi-shaped Ag NPs. For the DSSCs without metal NPs, the short-circuit current density (*J_sc_*), open-circuit voltage (*V_oc_*), fill factor (*ff*), and PCE were 15.86 mA/cm^2^, 0.76 V, 0.70, and 8.44%, respectively. By including spherical Ag NPs, the DSSCs with spherical Ag NPs showed the improvement of PCE. The photovoltaic parameters were 16.58 mA/cm^2^, 0.76 V, 0.69, and 8.69%, respectively. Compared to the DSSCs without metal NPs, the PCE of the DSSCs with spherical Ag NPs increased by 2.96% due to the first plasmon-enhanced effect. For the DSSCs with multi-shaped Ag NPs, the photovoltaic parameters were 16.73 mA/cm^2^, 0.75 V, 0.71, and 8.91%, respectively. Compared to the DSSCs with spherical Ag NPs, the PCE of the DSSCs with multi-shaped Ag NPs increased by 2.53% due to the plasmon-enhanced effect that covered the broad wavelength range in the λ_1_ of N719 dye. The total enhancement of DSSCs based on the layer-by-layer structure with multi-shaped Ag NPs increased from 8.44% to 8.91%, corresponding to a 5.57% enhancement.

To further improve the PCE of the DSSCs, the second plasmonic layer with Au NPs was introduced in the three types of the DSSCs based on the layer-by-layer structure. By including Au NPs, the PCE of the DSSCs was increased from 8.44% to 9.10% due to an increase in photocurrent density, from 15.86 mA/cm^2^ to 17.58 mA/cm^2^, by the plasmon-enhanced effect of the second plasmonic layer. For the DSSCs with spherical Ag and Au NPs, the photovoltaic parameters were 19.41 mA/cm^2^, 0.75 V, 0.68, and 9.90%, respectively. The PCE of the DSSCs with spherical Ag and Au NPs increased by 8.79% than those with Au NPs, because of the plasmon-enhanced effect of the first plasmonic layer. The photovoltaic parameters of the DSSCs with multi-shaped Ag and Au NPs were 19.76 mA/cm^2^, 0.75 V, 0.69, and 10.22%, respectively. When the DSSCs based on the layer-by-layer structure with multi-shaped Ag and Au NPs were compared to the DSSCs with spherical Ag and Au NPs, the PCE of the DSSCs with multi-shaped Ag and Au NPs increased by 3.23% due to the plasmon-enhanced effect that covered the broad wavelength range in the λ_1_ of N719 dye. The total enhancement of the DSSCs based on the layer-by-layer structure with multi-shaped Ag and Au NPs increased from 8.44% to 10.22%, corresponding to a 21.09% enhancement. This means that the PCE of the DSSCs with spherical Ag or multi-shaped Ag NPs was improved by the plasmon-enhanced effect, and the DSSCs with multi-shaped Ag NPs, which covered the broad wavelength range in the λ_1_ of N719 dye, exhibited better PCE than DSSCs with spherical Ag NPs. The dark current characteristics of DSSC based on the layer-by-layer structure without metal NPs, with spherical Ag and Au NPs, and with multi-shaped Ag and Au NPs were shown in Figure S5. In terms of dark current characteristics, *V_oc_* of the DSSC with spherical Ag or with multi-shaped Ag NPs was higher than that of the DSSC without metal NPs due to the improvement of electron density by spherical Ag or multi-shaped Ag NPs in the dark condition. 

**Figure 5:**
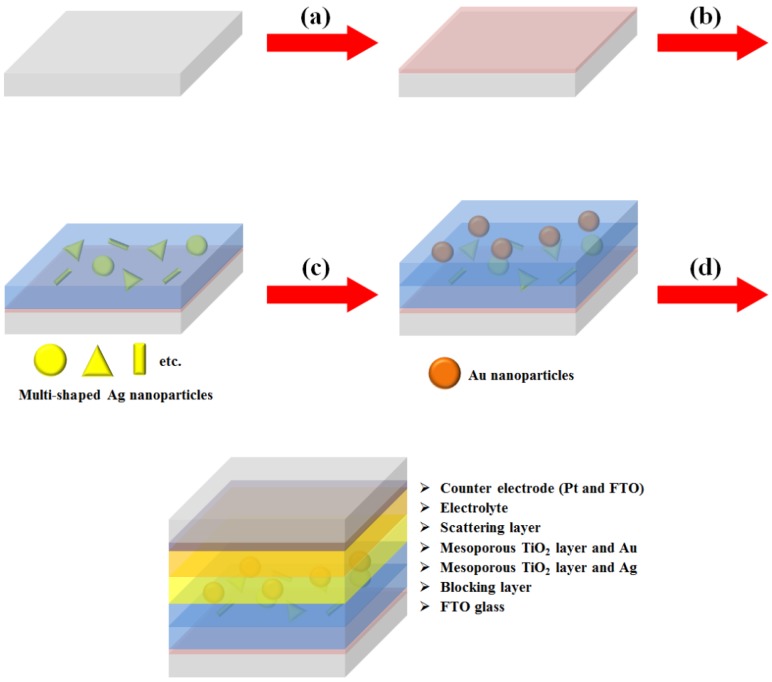
Fabrication process of dye-sensitized solar cells based on a composite film structure with multi-shaped Ag and Au nanoparticles (NPs): (**a**) coating of the TiO_2_ blocking layer, (**b**) coating of multi-shaped Ag NPs and mesoporous TiO_2_ NPs, (**c**) recoating of the Au NPs and mesoporous TiO_2_ NPs, and (**d**) fabrication of the DSSC.

Another type of DSSC fabricated with a composite film structure was used to confirm the plasmon-enhanced effect of the multi-shaped Ag NPs [18]. The fabrication of DSSCs composed of multi-shaped Ag and Au NPs is shown in Figure 5. Firstly, a TiO_2_ blocking layer was coated on FTO glass (Figure 5a). To prepare the first plasmonic layer for the λ_1_ of the N719 dye at 393 nm in the composite film structure of DSSCs, the multi-shaped Ag and mesoporous TiO_2_ NPs were mixed and coated on the TiO_2_ blocking layer using the doctor-blade technique (Figure 5b). To prepare the second plasmonic layer for the λ_2_ of the N719 dye at 533 nm in the composite film structure of DSSCs, the Au and mesoporous TiO_2_ NPs were mixed and coated on the first plasmonic layer using the doctor-blade technique (Figure 5c). Finally, the scattering layer was coated using the doctor-blade technique, and the DSSCs were fabricated with an electrolyte and a counter-electrode (Figure 5d).

**Figure 6:**
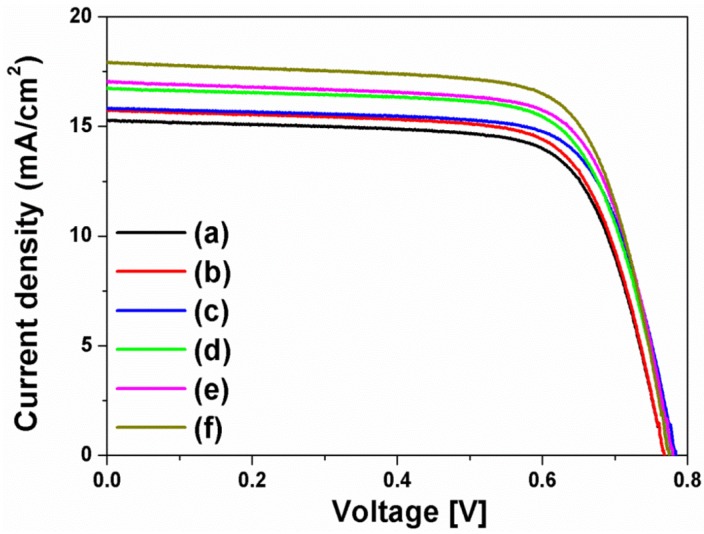
Current density-voltage curves of DSSCs based on a composite film structure: (**a**) without metal NPs, (**b**) with spherical Ag NPs, (**c**) with multi-shaped Ag NPs, (**d**) with Au NPs, (**e**) with spherical Ag and Au NPs, and (**f**) with multi-shaped Ag and Au NPs.

The I-V curves (Figure 6) were recorded for DSSCs based on the composite film structure with spherical Ag NPs, multi-shaped Ag NPs, or Au NPs and the photovoltaic parameters are summarized in Table 2. To study the plasmon-enhanced effect of the first plasmonic layer with spherical Ag or multi-shaped Ag NPs, excluding the second plasmonic layer, three types of DSSCs based on the composite film structure were fabricated without metal NPs, with spherical Ag NPs, or with multi-shaped Ag NPs. By including spherical Ag or multi-shaped Ag NPs, the PCE of the DSSCs was improved. Compared to the DSSCs based on the composite film structure without metal NPs, the PCE of those with spherical Ag NPs increased by 3.03% due to the plasmon-enhanced effect of the first plasmonic layer. And, the PCE of DSSCs with multi-shaped Ag NPs increased by 1.81% than those with spherical Ag NPs due to the plasmon-enhanced effect, which covered the broad wavelength range in the λ_1_ of N719 dye. In total, the enhancement of the DSSCs based on the composite film structure with multi-shaped Ag NPs increased from 8.58% to 9.00%, corresponding to a 4.89% enhancement. 

To further improve the PCE of the DSSCs with the first plasmonic layer using spherical Ag or multi-shaped Ag NPs, the second plasmonic layer with Au NPs was introduced in the three types of DSSC based on the composite film structure. By including Au NPs, the PCE of the DSSCs based on the composite film structure with Au NPs increased by 8.86% than those without metal NPs due to the second plasmon-enhanced effect. For the DSSCs with spherical Ag and Au NPs, the photovoltaic parameters were 17.07 mA/cm^2^, 0.78 V, 0.75, and 9.99%, respectively. The PCE of the DSSCs with spherical Ag and Au NPs increased by 6.96% than those with Au NPs because of the plasmon-enhanced effect of the first plasmonic layer. The photovoltaic parameters of the DSSCs with multi-shaped Ag and Au NPs were 17.91 mA/cm^2^, 0.78 V, 0.74, and 10.34%, respectively. When the DSSCs based on the composite film structure with multi-shaped Ag and Au NPs were compared to the DSSCs with spherical Ag and Au NPs, the PCE of DSSCs with multi-shaped Ag and Au NPs increased by 3.50% due to the plasmon-enhanced effect, which covered the broad wavelength range in the λ_1_ of N719 dye. In total, the enhancement of the DSSCs based on the composite film structure with multi-shaped Ag and Au NPs increased from 8.58% to 10.34%, corresponding to an improvement of 20.51%. Similar to the layer-by-layer structure, the PCE of the composite film structure in DSSCs with spherical Ag NPs or multi-shaped Ag NPs was improved by the plasmon-enhanced effect of the first plasmonic layer, and the DSSCs with multi-shaped Ag NPs, which have broader absorption wavelengths range in the λ_1_ of N719 dye, exhibited better PCE than the DSSCs with spherical Ag NPs.

Compared with DSSCs based on the layer-by-layer structure and based on the composite film structure, the *J_sc_* and *V_oc_* are different. Kamat’s group reported that there are two kinds of plasmonics; “plasmonic effect” and “charging effect” [9]. When DSSCs were fabricated with the layer-by-layer structure, the spherical Ag or multi-shaped Ag NPs were introduced onto the TiO_2_ film. In this case, the most electrons are transferred to TiO_2_ that changes *J_sc_*. So the main role of spherical Ag or multi-shaped Ag NPs in DSSCs based on the layer-by-layer structure is the light harvesting that is similar to “plasmonic effect”. However, when DSSCs were fabricated with the composite film structure, the spherical Ag or multi-shaped Ag NPs were mixed with TiO_2_ films. In this case, some electrons are transferred to TiO_2_ NPs, spherical Ag NPs, or multi-shaped Ag NPs that change the electron density and Fermi level on the TiO_2_ composite film structure. When electrons are transferred to the TiO_2_ NPs, the *J_sc_* will be changed. However, when electrons are transferred to spherical Ag or multi-shaped Ag NPs, the Fermi level has a more negative potential than that reflected to the *V_oc_* and the electron density is greater than that also reflected to *ff*. Thus, the main role of spherical Ag or multi-shaped Ag NPs in DSSCs based on the composite film structure is the improvement of electron density, which is similar to the “charging effect”.

**Figure 7:**
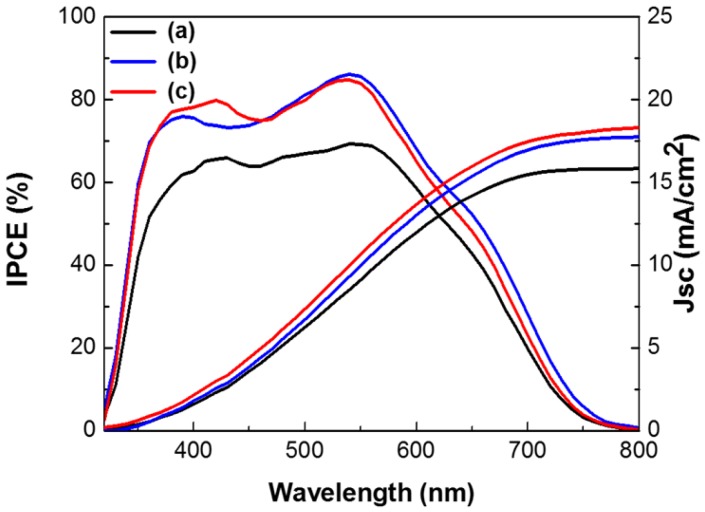
IPCE spectra and integrated current density of the DSSCs based on the layer-by-layer structure: (**a**) without metal NPs, (**b**) with spherical Ag and Au NPs, and (**c**) with multi-shaped Ag and Au NPs.

Figure 7 shows the incident photon-to-electron conversion efficiency (IPCE) and integrated current densities of DSSCs based on the layer-by-layer structure without metal NPs, with spherical Ag and Au NPs, or with multi-shaped Ag and Au NPs. In IPCE of the DSSCs, the intensity of the DSSCs without metal NPs was lower than those with Ag and Au NPs. In the DSSCs with Au NPs, the light absorption peak at 540 nm was higher and followed the same pattern. However, in the DSSCs with spherical Ag NPs or multi-shaped Ag NPs, the light absorption peaks were also higher, but followed different patterns. The IPCE of the DSSCs with spherical Ag NPs had a narrow light absorption peak at 390 nm, matching the UV-VIS spectrum of spherical Ag NPs. Meanwhile, the IPCE of the DSSCs with multi-shaped Ag NPs had a broad light absorption peak at 420 nm matching the UV-VIS spectrum of multi-shaped Ag NPs. This means that not only is plasmonic enhancement dependent on the light absorption of metal NPs, but also that the efficiency is improved by more electron generation within these wavelength ranges. Regarding the IPCE, both of the maximum light absorption peaks from the DSSCs were related to Ag NPs in the first plasmonic layer and Au NPs in the second plasmonic layer. The second plasmonic layer with Au NPs showed the same patterned light absorption peaks at 540 nm in the DSSCs with spherical or multi-shaped Ag NPs. The first plasmonic layer of the DSSCs with spherical Ag NPs showed a narrow light absorption peak, while that of the DSSC with multi-shaped Ag NPs showed a broad light absorption peak following a similar pattern to the UV-VIS spectra shown in Figure 3. This means more electrons were generated from N719 dye due to the light absorption of spherical Ag NPs, affecting the PCE of the DSSCs. The integrated current density of DSSCs based on the layer-by-layer structure without metal NPs, with spherical Ag and Au NPs, or with multi-shaped Ag and Au NPs from the IPCE spectra are 15.83, 17.76, and 18.30 mA/cm^2^, respectively, as shown in Figure 7. There is difference between these values and the *J_sc_* obtained from the I-V curves. Due to the difference in the measurement of I-V and IPCE, the difference between *J_sc_* from the I-V curve and the integrated current density from the IPCE spectra is generally observed. However, both of them exhibited a similar increasing tendency.

## 3. Materials and Methods

### 3.1. Synthesis of Multi-Shaped Ag NPs

The multi-shaped Ag NPs were prepared using a one-step seed-mediated process. To prepare the Ag seed solution, 0.30 mL silver nitrate (AgNO_3_, 10 mM) and trisodium citrate (1 mM) solutions were mixed by stirring for 5 min. Subsequently, 1.8 mL of sodium borohydride (NaBH_4_, 10 mM) was injected into the mixed solution, which was stirred for 5 min. To grow the multi-shaped Ag NPs, 4 mL of the Ag seed solution, 16 mL trisodium citrate solution, and 0.2 mL ascorbic acid (20 mM) solution were mixed. Then, 0.80 mL AgNO_3_ solution was injected into this solution, and the mixture was stirred for 30 min. 

### 3.2. Synthesis of Spherical Ag NPs

Spherical Ag NPs were synthesized via a previously reported method [22]: 9 mL of the Ag seed solution and 11 mL distilled water were mixed, and 1.2 mL sodium ascorbate (20 mM) solution was injected into the mixed solution. To this solution, 1.2 mL AgNO_3_ (10 mM) solution was rapidly injected, and the mixture was stirred vigorously for 15 min. 

### 3.3. Synthesis of Au NPs

Au NPs were synthesized via the Turkevich method [23]: 50 mL of HAuCl_4_ (0.5 mM) solution was heated to boiling point, and 300 μL of a 1% sodium citrate solution was injected into the heated HAuCl_4_ solution. The color of the mixture changed from purple to red, and the solution was stirred at its boiling point for 30 min. Subsequently, the Au colloid solution was cooled slowly by stirring.

### 3.4. Fabrication of DSSCs Based on Layer-By-Layer Structure

A TiO_2_ blocking layer was deposited on washed FTO glass by spin-coating it with 5 wt% of titanium di-isopropoxide bis(acetylacetonate) in butanol, which was annealed at 450 °C for 30 min. The mesoporous TiO_2_ layer was prepared from TiO_2_ (T/SP, Solaronix SA, Switzerland) using the doctor-blade technique and annealed at 450 °C for 30 min. P4VP was coated on the mesoporous TiO_2_ layer by dipping it in a P4VP solution (0.15 g P4VP in 50 mL ethanol). To immobilize the spherical Ag NPs or multi-shaped Ag NPs on the mesoporous TiO_2_ layer, TiO_2_-film-coated P4VP was dipped in Ag or multi-shaped Ag nanoparticle solutions. The mesoporous TiO_2_ layer was further coated using the doctor-blade technique, and P4VP was recoated on the mesoporous TiO_2_ layer. Au NPs were subsequently immobilized with P4VP on the mesoporous TiO_2_ layer by dipping it in the Au nanoparticle solution. After drying, the scattering layer (DSL 18NR-AO, Dyesol-Timo, Queanbeyan, NSW, Australia) was doctor-bladed and annealed at 450 °C for 30 min. The resulting film was treated by titanium isopropoxide solution (0.1 M in isopropyl alcohol) at 90 °C for 30 min and then annealed at 450 °C for 30 min. The films, including the metal NPs, were additionally annealed in inert gas contained with hydrogen at 450 °C for 10 min. The photoanode was immersed overnight in a N719 (Solaronix) dye solution in ethanol at 50 °C. Finally, the photoanode and Pt counter electrode were sandwiched by Surlyn. Subsequently, electrolyte was injected between the photoanode and the Pt counter electrode. The electrolyte was composed of 0.7 M 1-butyl-3-methylimidazolium iodide, 0.03 M I_2_, 0.1 M of guanidinium thiocyanate, and 0.5 M 4-tert-butylpyridine in a mixture of acetonitrile and valeronitrile (85:15 *v*/*v*). The active area of the DSSCs was 0.25 cm^2^.

### 3.5. Fabrication of DSSCs Based on Composite Film Structure

The fabrication process of the composite film structure was similar to that of the layer-by-layer structure. However, to prepare the photoanodes in the DSSCs with a composite film structure, mesoporous TiO_2_ nanoparticle paste was mixed with multi-shaped Ag NPs, or spherical Ag NPs (0.70 wt %) were coated on FTO glass using the doctor-blade technique. Mesoporous TiO_2_ nanoparticle paste was subsequently mixed with Au NPs (0.52 wt %), which were coated on the mesoporous TiO_2_ nanoparticle layer incorporated with multi-shaped Ag NPs or spherical Ag NPs. 

### 3.6. Characterization of DSSCs

The shape and size of the synthesized metal NPs were confirmed with an energy-filtering transmittance electron microscope (LIBRA 120, Carl Zeiss, Oberkochen, Germany). The UV-VIS absorption spectra of the Ag, multi-shaped Ag, and Au nanoparticle solutions were analyzed with UV-VIS absorption spectroscopy (Neosys-2000, Scinco, Seoul, Korea). The I-V characteristics of the DSSCs were measured using an electrometer (KEITHLEY 2400) under AM 1.5 illumination (100 mW/cm^2^) provided by a solar simulator (1 KW xenon with AM 1.5 filter, PEC-L01, Peccell Technologies, Inc., Yokohama, Japan). 

## 4. Conclusions

Multi-shaped Ag NPs, which had absorption wavelengths broader than those of spherical Ag NPs, were introduced to DSSCs to improve the PCE via the plasmon-enhanced effect. For both DSSCs with layer-by-layer and composite film structures, the PCEs of DSSCs with multi-shaped Ag NPs were better than those of DSSCs with spherical Ag NPs. This concept can be applied to improve the efficiency of perovskite solar cells, hybrid solar cells, and other solar cells devices.

## Figures and Tables

**Table 1:** Photovoltaic properties of DSSCs based on a layer-by-layer structure.

I-V curve	DSSCs Based on Layer-By-Layer Structure	*J_sc_* (mA/cm^2^)	*V_oc_* (V)	*ff*	*η* (%)
(a)	Without metal NPs	15.86	0.76	0.70	8.44
(b)	With spherical Ag NPs	16.58	0.76	0.69	8.69
(c)	With multi-shaped Ag NPs	16.73	0.75	0.71	8.91
(d)	With Au NPs	17.58	0.75	0.69	9.10
(e)	With spherical Ag NPs and Au NPs	19.41	0.75	0.68	9.90
(f)	With multi-shaped Ag NPs and Au NPs	19.76	0.75	0.69	10.22

**Table 2:** Photovoltaic properties of DSSCs based on a composite film structure.

I-V curve	DSSCs Based on Composite Film Structure	*J_sc_* (mA/cm^2^)	*V_oc_* (V)	*ff*	*η* (%)
(a)	Without metal NPs	15.27	0.77	0.73	8.58
(b)	With spherical Ag NPs	15.72	0.77	0.73	8.84
(c)	With multi-shaped Ag NPs	15.81	0.78	0.73	9.00
(d)	With Au NPs	16.72	0.78	0.72	9.34
(e)	With spherical Ag NPs and Au NPs	17.07	0.78	0.75	9.99
(f)	With multi-shaped Ag NPs and Au NPs	17.91	0.78	0.74	10.34

## Supplementary Materials

The following are available online at http://www.mdpi.com/2079-4991/7/6/136/s1, Figure S1: The cross-section image of SEM by photoanode in DSSC based on layer-by-layer structure. The thicknesses of the 1^st^ TiO_2_ layer (bottom), 2^nd^ TiO_2_ layer (middle) and scattering layer (top) were 5.44 μm, 4.83 μm, and 5.67 μm, respectively, Figure S2: The EDX spectra of (a) TiO_2_ film immobilized Au NPs, (b) TiO_2_ film immobilized Ag NPs and (c) TiO_2_ film immobilized Ag and Au NPs based on layer-by-layer structure, Figure S3: The SEM images and size distribution histograms of (a) immobilized Ag NPs and (b) immobilized Au NPs on film. The average size of Ag and Au NPs is 29 ± 1.8 nm and 19 ± 1.5 nm, respectively, Figure S4: Simulated electric field intensity distributions of (a) Au and (b) Ag NPs under the excitation at their plasmon wavelengths in layer-by-layer structure calculated by the finite difference time-domain (FDTD) method, Figure S5: Dark current characteristics of DSSCs based on the layer-by-layer structure (a) without metal nanoparticles (NPs), (b) with spherical Ag and Au NPs, and (c) with multi-shaped Ag and Au NPs.
