# Supplementary material for: Multi-Shaped Ag Nanoparticles in the Plasmonic Layer of Dye-Sensitized Solar Cells for Increased Power Conversion Efficiency
Source: Nanomaterials (Basel). 2017 Jun 4;7(6):136. doi: 10.3390/nano7060136 (PMC5485783; doi:10.3390/nano7060136)
Supplement: Supplementary file 1 [file nanomaterials-07-00136-s001.pdf]

Supporting Information

Multi-Shaped Ag Nanoparticles in the Plasmonic Layer of Dye-Sensitized Solar Cells for Increased Power Conversion Efficiency

Da Hyun Song[1], Ho-Sub Kim[1], Jung Sang Suh[1], Bong-Hyun Jun[2]*and Won-Yeop Rho[1]*

[1] Department of Chemistry, Seoul National University, Seoul 151-747, Republic of Korea; rho7272@gmail.com

[2] Department of Bioscience and Biotechnology, Konkuk University, Seoul 143-701, Republic of Korea; bjun@konkuk.ac.kr

* Correspondence: Bong-Hyun Jun: bjun@konkuk.ac.kr, Won-Yeop Rho: rho7272@gmail.com; Tel.: +82-2-450-0521; Fax. : +82-2-3437-1977

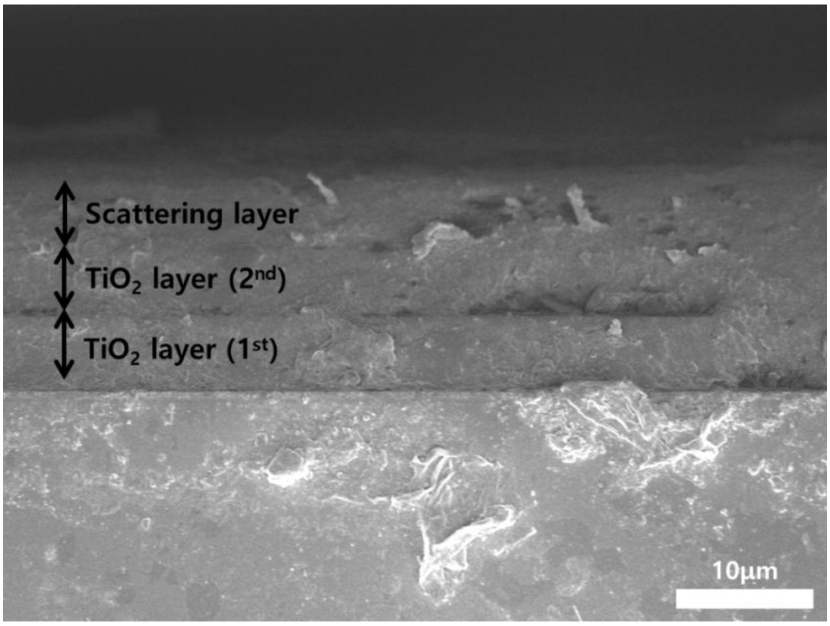

Figure S1. A SEM cross-section image of the photoanode in a dye-sensitized solar cell (DSSC) based on the layer-by-layer structure. The thicknesses of the first TiO_2 layer (bottom), second TiO_2 layer (middle), and scattering layer (top) were 5.44 μm, 4.83 μm, and 5.67 μm, respectively.

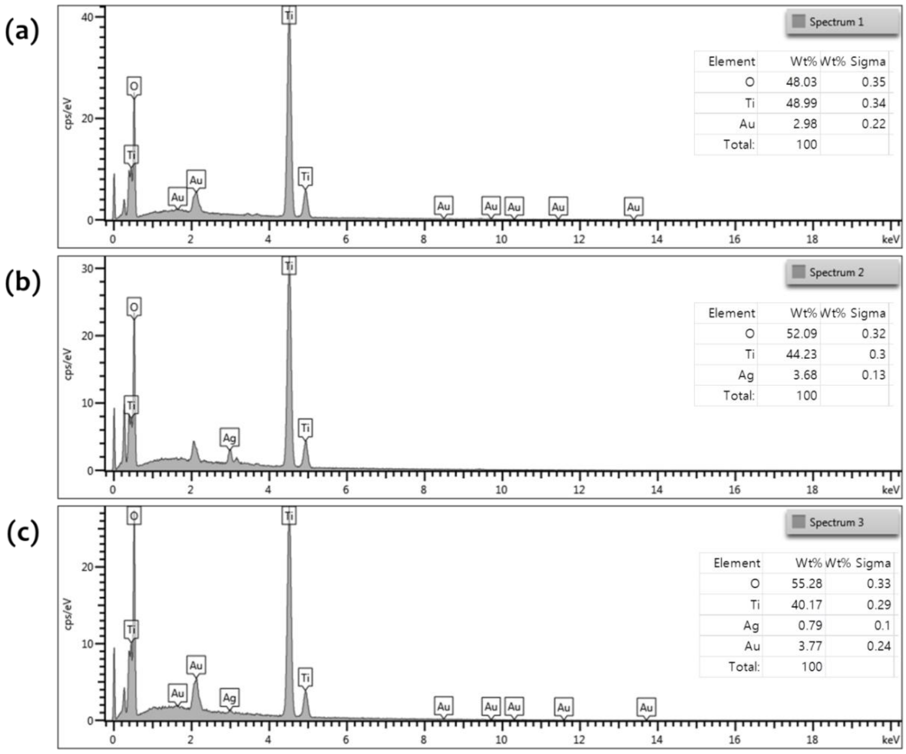

Figure S2. The EDX spectra of (**a**) TiO_2 film-immobilized Au NPs, (**b**) TiO_2 film-immobilized Ag NPs, and (**c**) TiO_2 film-immobilized Ag and Au NPs based on the layer-by-layer structure.

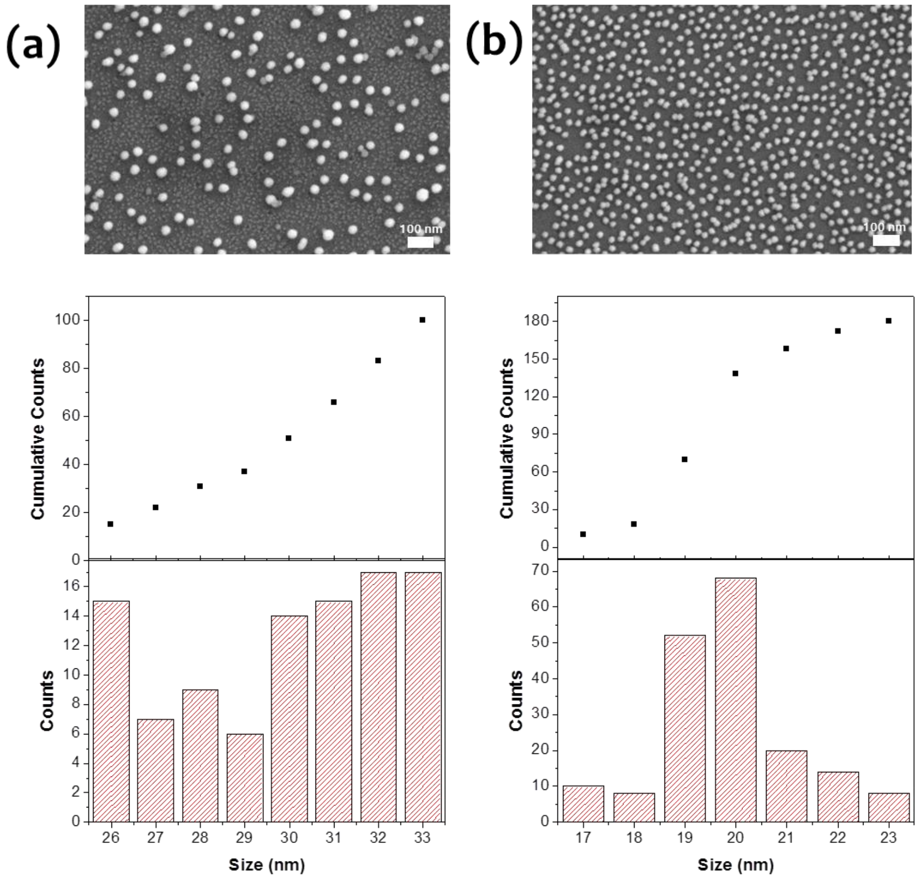

Figure S3. The SEM images and size distribution histograms of (**a**) immobilized Ag NPs and (**b**) immobilized Au NPs on film. The average size of Ag and Au NPs are 29±1.8 nm and 19±1.5 nm, respectively.

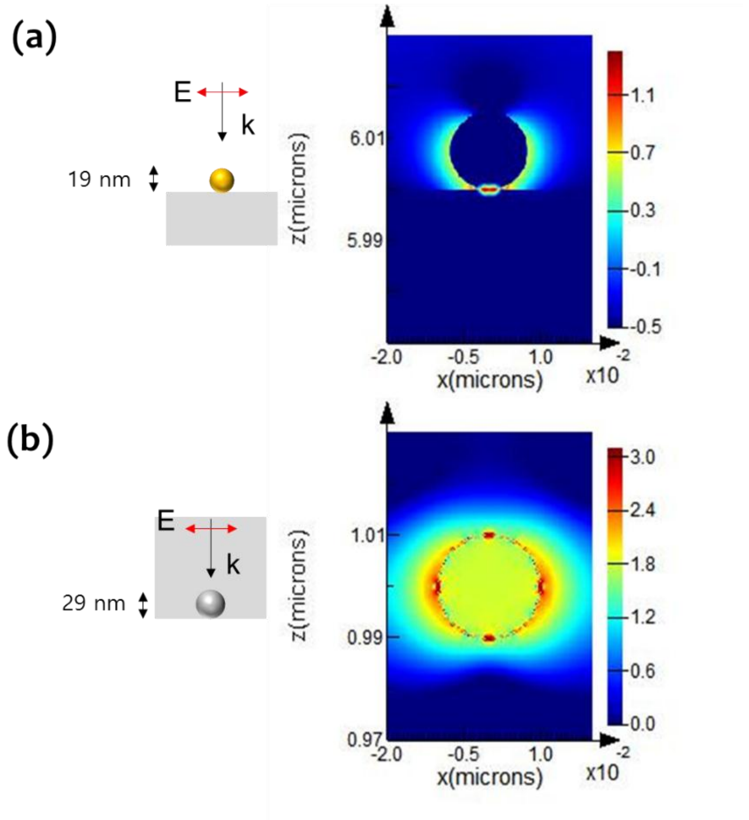

Figure S4. Simulated electric field intensity distributions of (**a**) Au and (**b**) Ag NPs under the excitation at their plasmon wavelengths in the layer-by-layer structure calculated by the finite difference time-domain (FDTD) method.

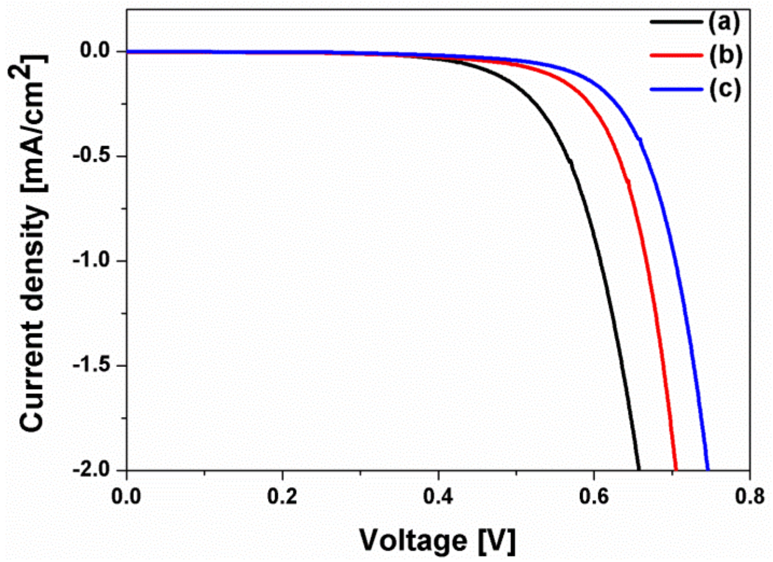

Figure S5. Dark current characteristics of DSSCs based on the layer-by-layer structure (**a**) without metal nanoparticles (NPs), (**b**) with spherical Ag and Au NPs, and (**c**) with multi-shaped Ag and Au NPs.

In terms of dark current characteristics, V_{oc} of the DSSC with Ag or with multi-shaped Ag nanoparticles was higher than that of the DSSC without metal nanoparticles. As shown in equation below, V_{oc} is related to J_{sc}, but not J_o, under one sun condition. This is because the electron density is increased by the light intensity, which changes the electron concentration and the Fermi level in the TiO_2 layer.

$$V_{oc} = \frac{nkT}{q} \ln\left(\frac{J_L}{J_o} + 1\right) \approx \frac{nkT}{q} \ln\left(\frac{J_L}{J_o}\right)$$

However, under dark conditions, Voc is also higher. This means than the electron density of the TiO2 layer is increased by Ag or multi-shaped Ag nanoparticles which also improved the diffusion length and electron lifetime. Thus, in our results, the Voc is improved due to the improvement of electron density by Ag or multi-shaped Ag nanoparticles under dark conditions.
